# Activation of arginine vasopressin receptor 1a reduces inhibitory synaptic currents at reciprocal synapses in the mouse accessory olfactory bulb

**DOI:** 10.3389/fncel.2024.1466817

**Published:** 2024-09-24

**Authors:** Mutsuo Taniguchi, Yoshihiro Murata, Masahiro Yamaguchi, Hideto Kaba

**Affiliations:** Department of Physiology, Kochi Medical School, Kochi University, Nankoku, Japan

**Keywords:** accessory olfactory bulb, vasopressin, patch clamp, granule cell, mitral cell

## Abstract

Central arginine vasopressin (AVP) facilitates social recognition and modulates many complex social behaviors in mammals that, in many cases, recognize each other based on olfactory and/or pheromonal signals. AVP neurons are present in the accessory olfactory bulb (AOB), which is the first relay in the vomeronasal system and has been demonstrated to be a critical site for mating-induced mate recognition (olfactory memory) in female mice. The transmission of information from the AOB to higher centers is controlled by the dendrodendritic recurrent inhibition, i.e., inhibitory postsynaptic currents (IPSCs) generated in mitral cells by recurrent dendrodendritic inhibitory inputs from granule cells. These reports suggest that AVP might play an important role in regulating dendrodendritic inhibition in the AOB. To test this hypothesis, we examined the effects of extracellularly applied AVP on synaptic responses measured from mitral and granule cells in slice preparations from 23-–36-day-old Balb/c mice. To evoke dendrodendritic inhibition in a mitral cell, depolarizing voltages of −70 to 0 mV (10 ms duration) were applied to a mitral cell using a conventional whole-cell configuration. We found that AVP significantly reduced the IPSCs. The suppressive effects of AVP on the IPSCs was diminished by an antagonist for vasopressin receptor 1a (V1aR) (Manning compound), but not by an antagonist for vasopressin receptor 1b (SSR149415). An agonist for V1aRs [(Phe^2^)OVT] mimicked the action of AVP on IPSCs. Additionally, AVP significantly suppressed voltage-activated currents in granule cells without affecting the magnitude of the response of mitral cells to gamma-aminobutyric acid (GABA). The present results suggest that V1aRs play a role in reciprocal transmission between mitral cells and granule cells in the mouse AOB by reducing GABAergic transmission through a presynaptic mechanism in granule cells.

## 1 Introduction

Arginine vasopressin (AVP) facilitates social recognition and modulates many complex social behaviors of mammals that depend on individual recognition (Terranova et al., 2017), including parental behavior, aggression, affiliation, pair bonding, and social memory (Caldwell et al., 2008; Wacker et al., 2011; Caldwell, 2012; Bosch, 2013; Freeman and Young, 2016; Numan and Young, 2016). In many cases, mammals distinguish each other based on olfactory and/or pheromonal signals.

**Figure 1:**
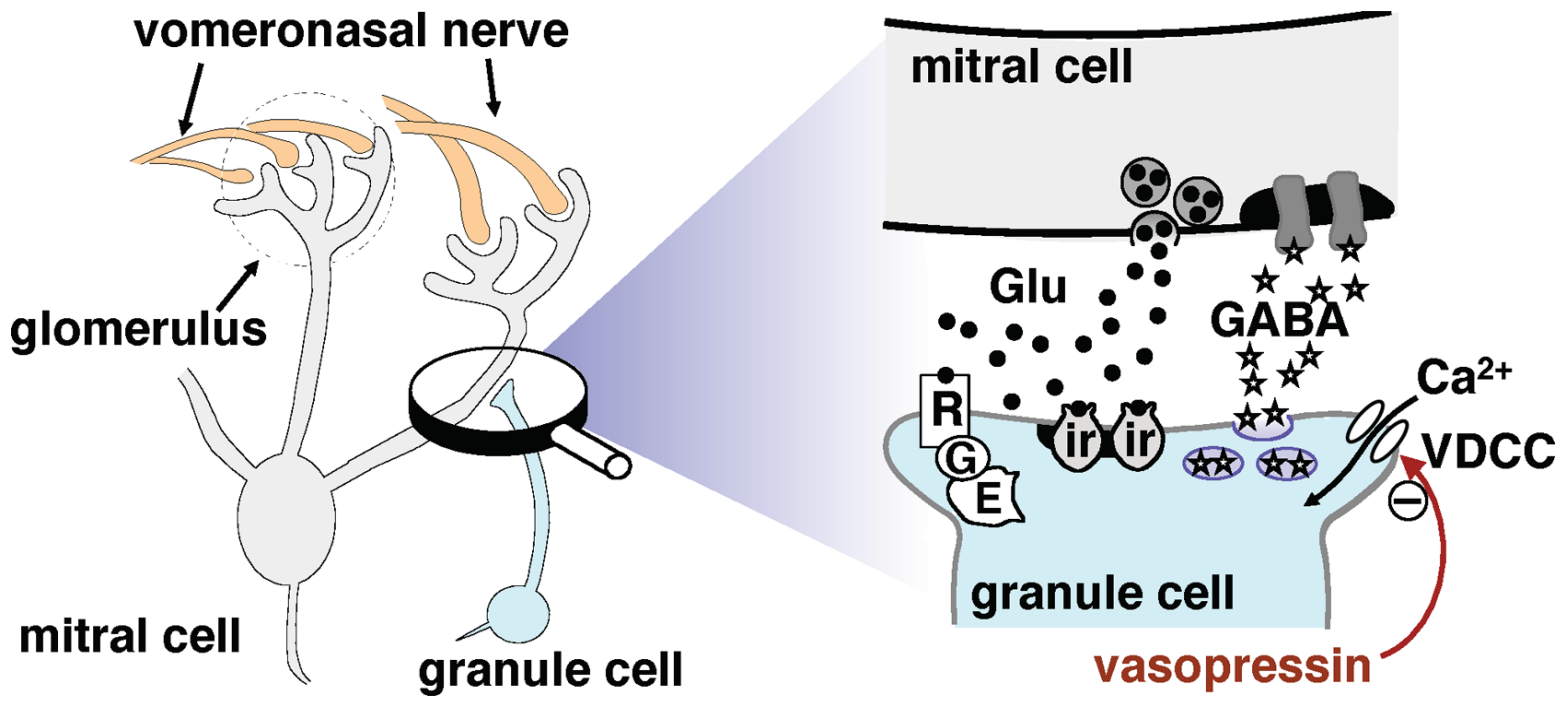
Synaptic organization potentially involved in dendrodendritic transmission between mitral and granule cells in the accessory olfactory bulb. For more details, see the main text. The signaling pathways that inhibit gamma-aminobutyric acid release are marked by a minus symbol; the underlying mechanisms remain unclear. Abbreviations: E, intracellular effector; G, G-protein; Glu, glutamate; ir, ionotropic glutamate receptor; R, mGluR2; VDCC, voltage-dependent calcium channel.

Pheromonal signals are mainly detected by vomeronasal sensory neurons, which send their axons to the accessory olfactory bulb (AOB); this is the first relay in the vomeronasal system. Newly mated female mice form long-lasting olfactory recognition memories of the pheromonal signals of mating males. These memories are vital for preventing the pregnancy failure that might otherwise be induced by a mate’s pheromones (Keverne and De la Riva, 1982; Brennan et al., 1990). The synaptic changes underlying these memories occur in the AOB (Kaba et al., 1989). Neurocircuits in the AOB involve reciprocal dendrodendritic synapses between mitral cells, the principal projection neurons, and granule cell interneurons. As mentioned above, mitral cells receive afferents from the vomeronasal nerve and project to the medial amygdala, forming an excitatory pathway to the hypothalamus for pheromonal signals received by vomeronasal receptor neurons (Halpern and Martinez-Marcos, 2003; Binns and Brennan, 2005; Brennan and Zufall, 2006) (partly illustrated in Figure 1). Mitral cell dendrites release glutamate to excite granule cell spines, which in turn provide feedback that inhibits mitral cells via the release of gamma-aminobutyric acid (GABA). The information flow from the AOB to higher centers is controlled by this dendrodendritic recurrent inhibition of mitral cells by granule cells.

Mice lacking a functional vomeronasal system show many of the behavioral deficits exhibited by mice that lack vasopressin receptor 1 (V1R). For example, male mice with an absence of pheromone-evoked neuronal activity in the vomeronasal organ, caused by a deficiency in transient receptor potential cation channel, subfamily C, member 2 expression, fail to display male–male aggression (Stowers et al., 2002). V1R consists of receptor type 1a (V1aR) and receptor type 1b (V1bR), which are seven-transmembrane G-protein-coupled receptors associated with phosphoinositol turnover. V1a is found in a variety of brain regions including the cerebellum, cerebral cortex, hippocampus, and hypothalamus (Ostrowski et al., 1994; Szot et al., 1994; Johnson et al., 1995; Tribollet et al., 1999). In contrast, V1bR expression is restricted to CA2 pyramidal neurons in the hippocampus (Young et al., 2006; Pagani et al., 2015). Genetic depletion of V1aRs (Egashira et al., 2007) or V1bRs (Wersinger et al., 2002, 2007; Stevenson and Caldwell, 2012) significantly reduces aggression in male mice. A study using a selective high-affinity V1aR antagonist (^125^I-sarc-AVP) showed that there are V1aRs on both mitral and granule cells in the AOB of microtine rodents, although subtle differences among species were apparent (Insel et al., 1994). These results, along with earlier studies of AVP’s effects on pair bonding, also suggest that AVP is an important mediator of social behaviors.

These reports suggest that AVP might play an important regulatory role in reciprocal synaptic transmission in the AOB. To test this hypothesis, we examined the effects of extracellularly applied AVP on reciprocal synaptic currents generated in AOB mitral cells. We used whole-cell patch-clamp recordings to investigate the mechanism and site of action of AVP. As shown in Figure 1, the reciprocal synapse is characterized by mitral-to-granule glutamatergic transmission and granule-to-mitral GABAergic transmission. The signals output from the AOB to higher centers are controlled by this dendrodendritic recurrent inhibition of mitral cells by granule cells. Therefore, in the present study, we focused on the effect of AVP on granule-to-mitral GABAergic transmission.

## 2 Materials and methods

### 2.1 Animals

All experiments were conducted in accordance with the guidelines of the Physiological Society of Japan and were approved by the Kochi Medical School Animal Care and Use Committee. Every effort was made to minimize the number of animals used and their suffering. The experimental animals were commercially available Balb/c mice (SLC, Hamamatsu, Japan).

### 2.2 Accessory olfactory bulb slice preparation

The methods used in this study are similar to those described previously (Taniguchi and Kaba, 2001). In brief, AOB slices were obtained from 23 to 36-day-old Balb/c female that were anesthetized with isoflurane and decapitated. After the AOB and main olfactory bulb had been dissected out, the olfactory bulbs were cut into slices ~300 μm thick with a vibrating slicer in ice-cold modified Ringer’s cutting solution containing (in mM) 200 sucrose, 3 KCl, 1 NaH_2_PO_4_, 25 NaHCO_3_, 0.5 CaCl_2_, 3.6 MgCl_2_, and 15 glucose (pH maintained at 7.4 by saturation with 95% O_2_—5% CO_2_). Parasagittal slices were incubated in a holding chamber for 60 min at 31°C and then transferred into recording chambers for conventional whole-cell recordings.

The preparations were viewed under an upright microscope (Axioskop 2 FS; Zeiss, Jena, Germany) with infrared differential interference contrast optics (filter, 850 nm) and a charge-coupled device camera (C2400; Hamamatsu Photonics, Hamamatsu, Japan) using a 40× water immersion lens (Zeiss). Mitral cells and internal granule cells could be discriminated easily on the basis of their morphology and location the middle and deep external plexiform layer and the internal cell layer, respectively (Larriva-Sahd, 2008).

### 2.3 Data recording and analysis

Membrane currents were recorded in the conventional whole-cell configuration (holding potential, −70 mV). For whole-cell configurations, patch pipettes with resistances of 5–7 MΩ were made from borosilicate glass capillaries (1B150F-4; WPI, Sarasota, FL, United States) using an electrode puller (P-97; Sutter Instruments, Novato, CA, USA) and then heat polished. Measurements were made using a patch-clamp amplifier (EPC9 Double; Heka, Lambrecht, Germany). Data were stored on a Macintosh personal computer. Membrane currents were recorded, filtered at 3 kHz, digitized at 1–20 kHz, and stored for off-line analysis, which was performed on a personal computer using Pulse and PulseFit software (Heka). To quantify dendrodendritic inhibition, we calculated the current integral over a 3-s period beginning 50–230 ms after the end of the voltage application according to the analysis method by other groups (Isaacson and Strowbridge, 1998; Schoppa et al., 1998; Maher and Westbrook, 2005). All recordings were performed at room temperature (23–26°C).

### 2.4 Statistical analysis

The data of all groups were evaluated for normality using the Shapiro–Wilk test, and for homogeneity of variance using Bartlett’s test. Standard parametric tests were performed only for data passing the normality and variance tests (*p* > 0.05); in all other cases, nonparametric tests were used. For comparison between groups using different drugs, repeated-measures analysis of variance with *post hoc* multiple contrasts were performed. Unless otherwise specified, all values are reported as mean ± standard error of the mean. In all analyses, *p* < 0.05 was considered statistically significant.

### 2.5 Solutions

The recording chamber was continuously perfused with normal Ringer’s solution consisting of (in mM) 125 NaCl, 3 KCl, 1 NaH_2_PO_4_, 25 NaHCO_3_, 2 CaCl_2_, 1 MgCl_2_, and 15 glucose (pH maintained at 7.4 via saturation with 95% O_2_—5% CO_2_). Tetrodotoxin (TTX), [*β*-mercapto-β, β-cyclopentamethylenepropionyl, o-me-Tyr2,Arg8]-vasopressin (Manning compound), (2S,4R)-1-[(R)-5-chloro-1-(2,4-dimethoxy-benzenesulfonyl)-3-(2-methoxy-phyenyl)-2-oxo-2,3-dihydro-1H-indol-3-yl]-4-hydroxy-pyrrolidine-2-carboxylic acid dimethylamide (SSR149415), and [Phe^2^, Orn^8^]-vasotocin [(Phe^2^)OVT] were dissolved in normal Ringer’s solution containing no Mg^2+^ to the desired final concentrations. All experiments were performed in the presence of TTX (1 μM). Measurements of granule cell calcium currents and the current response of mitral cells to GABA were made in the presence of 6-cyano-7-nitroquinoxaline-2,3-(1H,4H)-dione (CNQX; 10 µM) and D, L-2-amino-5-phosphonovaleric acid (AP5; 50 μM) to block glutamatergic transmission-mediated events and to isolate GABAergic transmission from granule to mitral cells. Patch pipettes were filled with Cs^+^ internal pipette solution containing (in mM) 140 CsCl, 5 NaCl, 1 MgCl_2_, 1 EGTA,10 HEPES, 2 Na_2_ATP, and 0.2 Na_3_GTP (pH 7.4). To isolate voltage-gated calcium currents, CsCl was decreased to 100 mM, and tetraethylammonium chloride (TEA-Cl) (30 mM) and lidocaine N-ethyl bromide (QX-314, 5 mM) were added (TEA^+^ internal pipette solution).

Gravity was used to deliver a constant stream of Ringer’s solution from the stimulating tube. Four electrically actuated valves were used to switch from normal Ringer’s solution to a conditioning extracellular solution. The stimulating tube, which had a lumen ~200 μm in diameter, was placed under visual control within ~500 μm of the mitral cell. An extracellular solution including desired chemical(s) were applied through this stimulating tube at the same flow rate as that of the solution used under the control condition. The concentrations of the chemicals were indicated by the concentrations in the stimulating tube.

### 2.6 Chemicals

TTX, SSR149415, and [Phe^2^] OVT were purchased from Wako Pure Chemical Industries, Ltd. (Osaka, Japan), Axon MedChem LLC (Reston, VA, United States) and Phoenix Pharmaceuticals, Inc. (Burlingame, CA, United States), respectively. AVP, Manning compound, QX-314, CNQX, and AP5 were purchased from Sigma Chemical Co. (St. Louis, MO, United States). All other reagents were of the highest grade commercially available.

## 3 Results

### 3.1 Arginine vasopressin suppresses dendrodendritic inhibition in the AOB

As mentioned in the Introduction section, the flow of information from the AOB to higher centers is controlled by the dendrodendritic recurrent inhibition of mitral cells by granule cells. We first examined whether AVP has an effect on reciprocal synaptic currents triggered by endogenous glutamate release from mitral cells. To address this question, we initially examined the effect of this hormone on reciprocal transmission between mitral and granule cells by stimulating a mitral cell and recording the evoked IPSCs.

In our experiments, the concentration of Cl^−^ was almost the same between the bath and the internal solutions, such that the reversal potential for GABA_A_ receptor-mediated current responses was ~0 mV. IPSCs should be recorded as inward currents at negative holding potentials. To evoke dendrodendritic inhibition, depolarizing voltage was applied to a mitral cell with CsCl-based internal solution (Cs^+^ internal pipette solution), from a holding potential of −70 to up 0 mV (10 ms), to increase the excitability of the cell and thus allow for adequate IPSCs. In all experiments, no Mg^2+^ was added to the extracellular solutions because activation of the N-methyl-D-aspartic acid (NMDA) receptor on mitral cells is important for dendrodendritic inhibition (Taniguchi and Kaba, 2001). As described in the Materials and Methods section, all experiments were performed in the presence of TTX to block Na^+^ channels and thus prevent any effect of axonal transmission.

**Figure 2:**
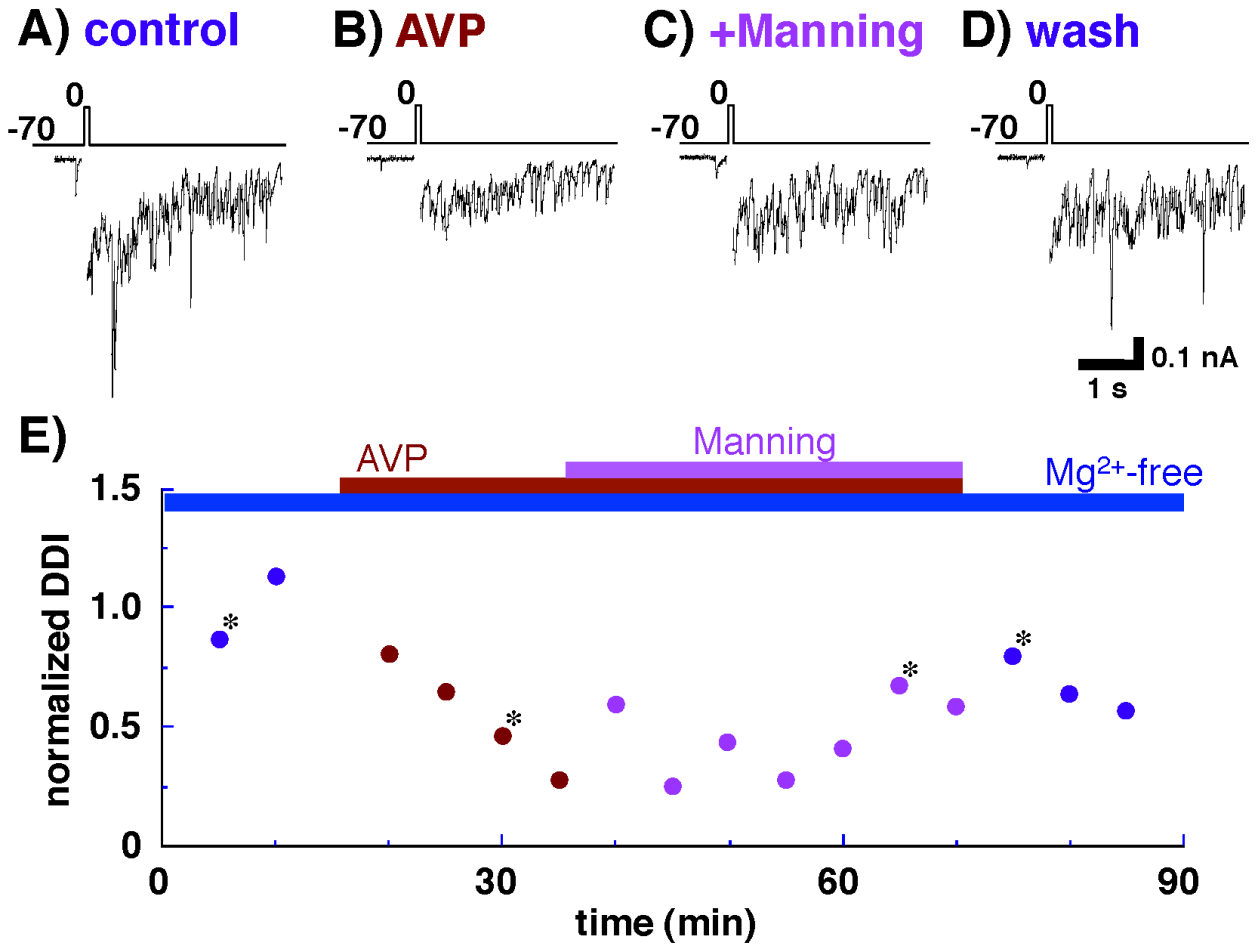
Whole-cell recording of a mitral cell with Cs^+^ internal pipette solution. Arginine vasopressin (AVP) significantly reduced inhibitory postsynaptic currents (IPSCs) through vasopressin receptor 1a (V1aR). IPSCs were evoked by applying depolarizing voltage to a mitral cell from a holding potential of −70 to up 0 mV (10 ms duration) as shown in the top traces. **(A)** IPSCs recorded from a mitral cell bathed in Ringer’s solution containing no Mg^2+^. **(B)** IPSCs recorded from the same cell bathed in Ringer’s solution containing AVP (0.2 nM) and no Mg^2+^. **(C)** IPSCs observed after the addition of a V1aR antagonist (Manning compound; 1 nM). **(D)** IPSCs observed after the removal of AVP and Manning compound. **(E)** Normalized magnitudes of dendrodendritic inhibition (DDI) evoked by voltage application. The magnitude of DDI was calculated relative to the mean magnitude with Ringer’s solution containing no Mg^2+^. To illustrate the synaptic component of the evoked responses more clearly, the calcium current generated during voltage application was blanked. Bars above the scatter graph indicate periods of extracellular application of tetrodotoxin, Mg-free Ringer’s solution, AVP, and Manning compound. The asterisks indicate the time points at which traces in part labels **A–D** were successively taken. All traces **(A–D)** and data points **(E)** were obtained from the same cell.

Under these conditions, depolarizing voltages, reported to evoke a relatively slow inward calcium current (Isaacson and Strowbridge, 1998; Schoppa et al., 1998; Taniguchi and Kaba, 2001; Castro et al., 2007; Taniguchi et al., 2013), were applied, followed by IPSCs (Figure 2A). AVP (0.2 nM) application markedly reduced dendrodendritic inhibition from 300 ± 61 pA•s to 138 ± 14 pA•s (46.0 ± 4.7% of the control; *n* = 7, *p* = 0.037, paired *t*-test) (Figure 2B). There are two well-characterized receptors for AVP in the brain: the V1aR and the V1bR (for a review, see Rigney et al., 2022). To determine the contribution of different AVP receptor subtypes to the reduced IPSCs, an antagonist of the V1aR was applied (Figure 2C). The reduced dendrodendritic inhibition following AVP application was restored to 279 ± 46 pA•s (93.0 ± 15.3% of the control; n = 7, *p* = 0.793, paired *t*-test) by the application of Manning compound. These results suggest that AVP plays a role in reciprocal dendrodendritic transmission between mitral cells and granule cells in the AOB through V1aRs.

**Figure 3:**
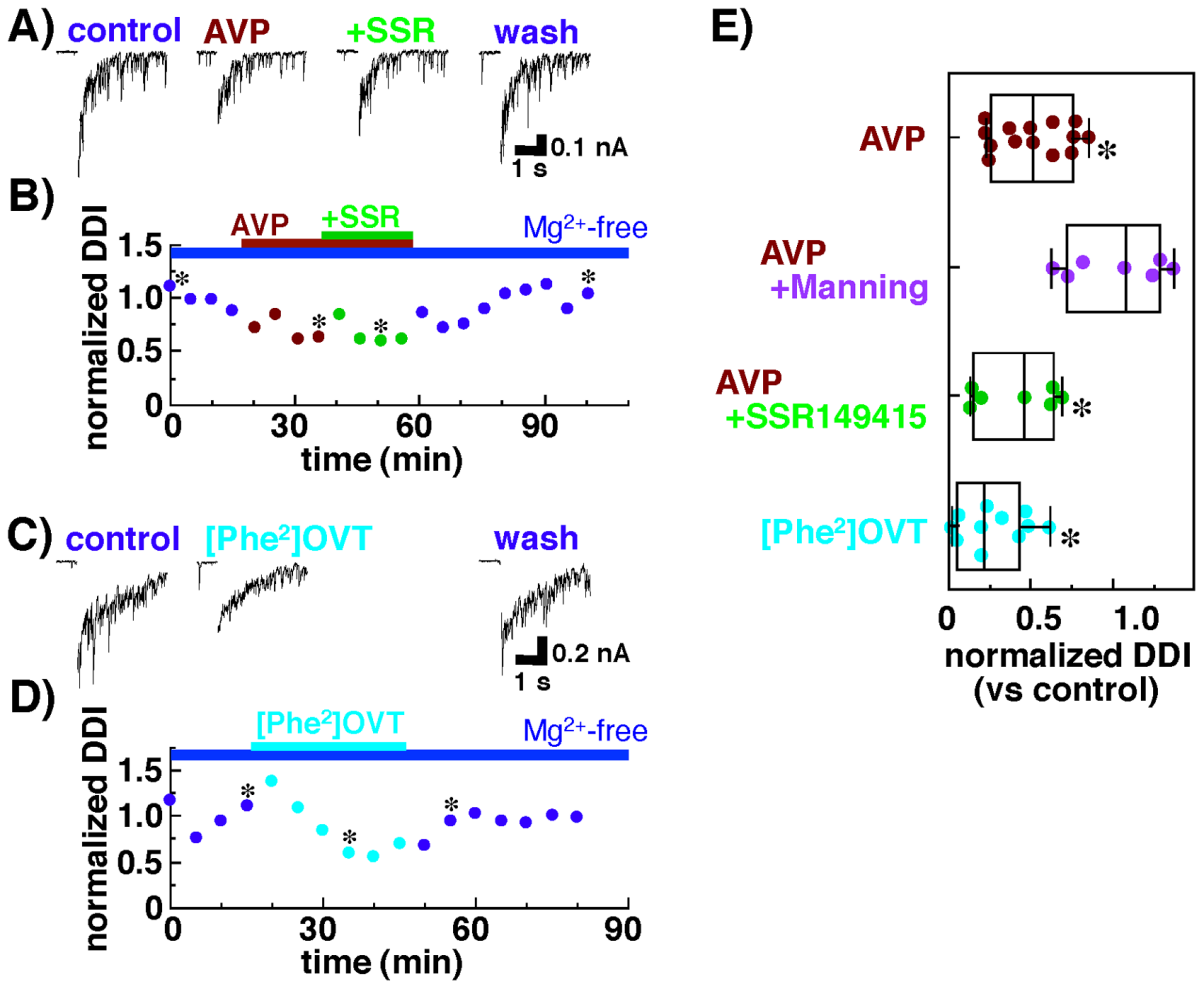
Summary of the effects of an agonist and an antagonist of arginine vasopressin (AVP) receptors on the dendrodendritic inhibition of mitral cells bathed in Ringer’s solution containing no Mg^2+^ with Cs^+^ internal pipette solution. **(A)** Inhibitory postsynaptic currents (IPSCs) recorded from a mitral cell before and during the application of AVP (0.2 nM) with or without an antagonist for the vasopressin receptor 1b, SSR149415 (100 nM). **(B)** Normalized magnitudes of dendrodendritic inhibition (DDI) evoked by voltage application in the same cell in **(A)**. The magnitude of DDI was calculated relative to the mean magnitude with Ringer’s solution containing no Mg^2+^. To illustrate the synaptic component of the evoked responses more clearly, the calcium current generated during voltage application was blanked. Bars above the scatter graph indicate periods of extracellular application of Mg-free Ringer’s solution and respective drugs. The asterisks indicate the time points at which traces in **(A)** were successively taken. **(C)** IPSCs recorded from a mitral cell before and during the application of an agonist for the vasopressin receptor 1a (V1aR), [Phe^2^, Orn^8^]-vasotocin (0.6 nM). **(D)** Normalized magnitudes of dendrodendritic inhibition (DDI) evoked by voltage application in the same cell in **(C)**. Bars above the scatter graph indicate periods of extracellular application of Mg-free Ringer’s solution and the drug. The asterisks indicate the time points at which traces in **(C)** were successively taken. **(E)** Normalized magnitude of dendrodendritic inhibition recorded in the presence of an agonist or an antagonist of AVP receptors. Each dot represents one mitral cell. The lines inside the boxes denote the median, the box boundaries indicate the 25th and 75th percentiles, and the whiskers indicate the range (minimum to maximum). In Mg^2+^-free solution, Manning compound significantly diminished the suppressive effect of AVP, whereas SSR149415 had almost no effect on the action of AVP (*p < 0.05 vs. control, Tukey–Kramer’s test). Consistent with these results, the effects of AVP on dendrodendritic inhibition were mimicked by the agonist for the V1aR.

We also examined the contribution of V1bRs to the decreased dendrodendritic inhibition recorded from mitral cells. As shown in Figures 3A,B, dendrodendritic inhibition was reduced by the application of AVP (0.2 nM) from 833 ± 228 pA•s to 324 ± 57 pA•s (38.9 ± 6.8% of the control; n = 7, *p* = 0.038, paired *t*-test). Unlike the antagonist of V1aRs, however, additional application of SSR149415 did not affect the amplitude of dendrodendritic inhibition (277 ± 59 pA•s; 33.3 ± 7.1% of the control; *n* = 7, *p* = 0.036, paired *t*-test). These results suggest that V1bRs have little effect on reciprocal dendrodendritic transmission between mitral cells and granule cells in the AOB.

Next, we tested whether dendrodendritic inhibition was affected by an agonist for V1aRs, [Phe^2^, Orn^8^]-vasotocin (0.6 nM). Dendrodendritic inhibition was reduced from 1,325 ± 384 nA•s to 310 ± 91 pA•s (23.4 ± 6.9% of the control; *n* = 11, *p* = 0.026, paired *t*-test) after [Phe^2^, Orn^8^]-vasotocin application (Figures 3C,D).

Figure 3E summarizes the effects of the agonist for V1aRs and the antagonist for V1aRs and V1bRs on dendrodendritic inhibition; the effects were mediated by reciprocal synapses between mitral and granule cells in the AOB. The data indicate that the suppressive effect of AVP on dendrodendritic inhibition was blocked not by the antagonist for V1bRs but, rather, by the antagonist for V1aRs, and the effect of AVP was mimicked by the agonist for V1aRs. These results suggest that AVP attenuates reciprocal transmission between mitral and granule cells through V1aRs.

### 3.2 Arginine vasopressin can modulate GABAergic transmission from granule to mitral cells in the AOB

Reciprocal transmission encompasses both glutamatergic transmission from mitral to granule cells and GABAergic transmission from granule to mitral cells. Thus, it is unclear whether the reduction in dendrodendritic inhibition induced by AVP is due to the suppression of mitral-to-granule cell excitatory transmission and/or the suppression of granule-to-mitral cell GABAergic transmission. In the present study, we focused on the effect of AVP on GABAergic transmission because of its direct influence on the membrane excitability of mitral cells, which serve as output neurons in the AOB projecting to higher centers.

**Figure 4:**
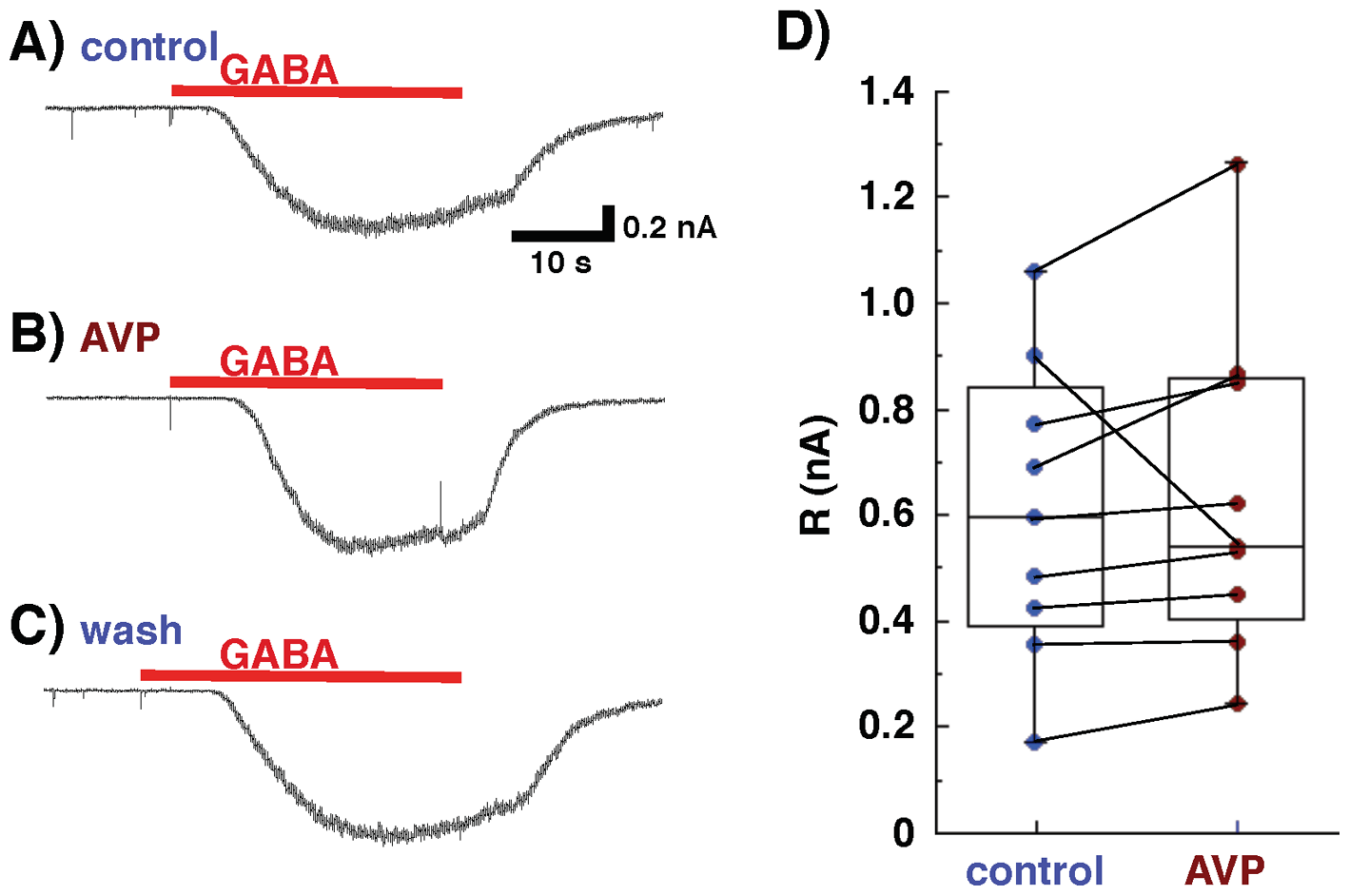
Whole-cell recording from mitral cells, using Cs^+^ internal pipette solution in the presence of 6-cyano-7-nitroquinoxaline-2,3-(1H,4H)-dione and D, L-2-amino-5-phosphonovaleric acid to block glutamatergic transmission. Arginine vasopressin (AVP) did not affect the response of mitral cells to gamma-aminobutyric acid (GABA). Response to GABA (10 μM) of a mitral cell bathed in Ringer’s solution containing no Mg^2+^ before [**(A)**: control], during [**(B)** AVP], and after [**(C)** wash] the application of AVP (0.2 nM). **(D)** Data on current responses to GABA pooled from nine experiments. The lines inside the boxes denote medians, the box boundaries indicate the 25th and 75th percentiles, and the whiskers indicate the range (minimum to maximum).

To investigate directly the effect of AVP on postsynaptic GABAergic transmission in mitral cells, the current responses of mitral cells to GABA application were recorded in the presence of antagonists for glutamatergic transmission, CNQX (10 μM) and AP5 (50 μM). Bath application of GABA (10 μM) elicited a current response in mitral cells (Figure 4A). Extracellular application of AVP did not affect the magnitude of the response of mitral cells to GABA at a concentration of 10 μM (606 ± 93 pA for the control, 637 ± 10 pA with APV application [105 ± 2% of the control], *p* = 0.586, *n* = 9, paired t-test; Figures 4B,D), suggesting that APV modulates the transmission from granule to mitral cells not through a postsynaptic but, rather, through a presynaptic mechanism in granule cells.

**Figure 5:**
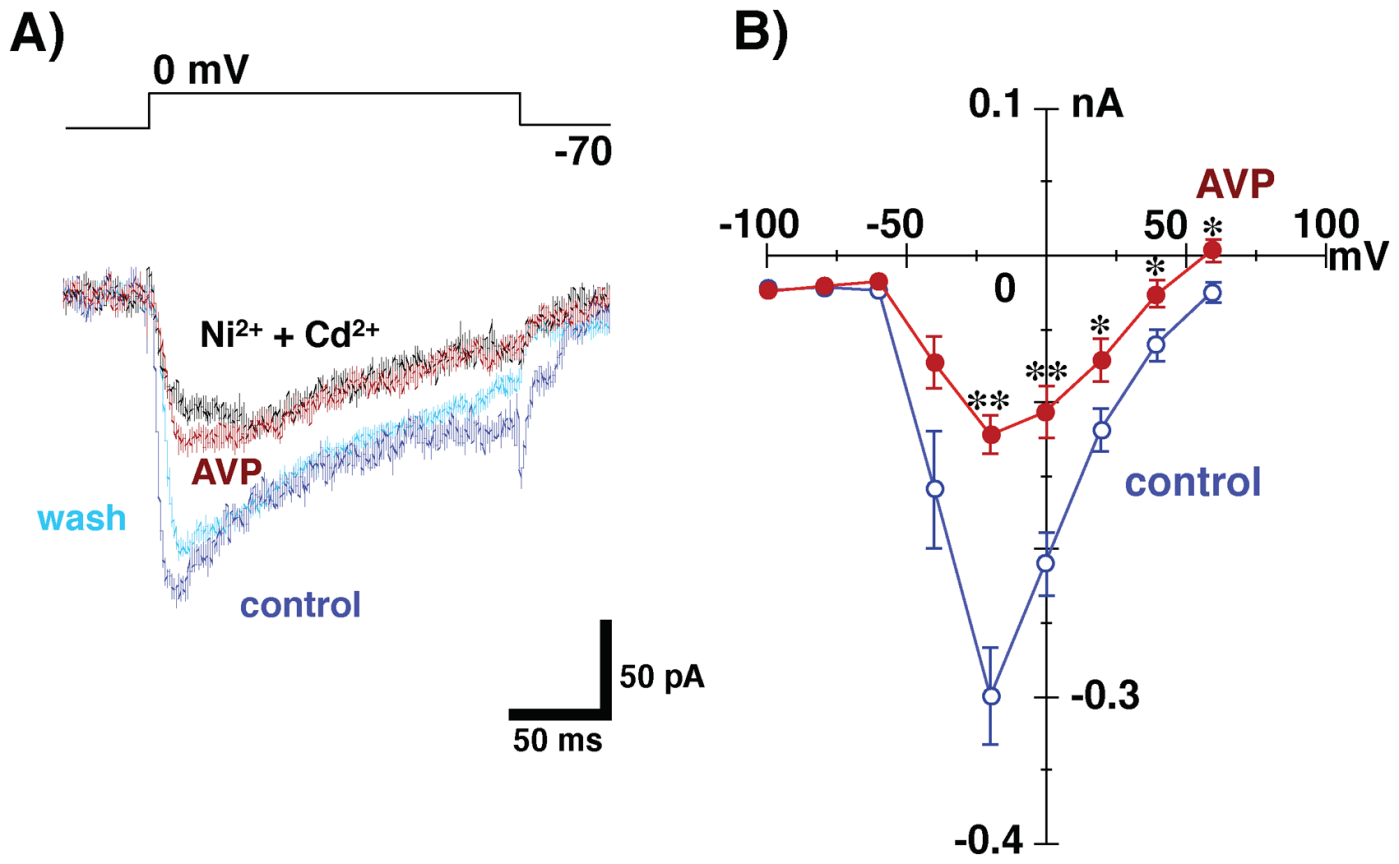
Whole-cell recording from granule cells using tetraethylammonium internal pipette solution in the presence of 6-cyano-7-nitroquinoxaline-2,3-(1H,4H)-dione and D, L-2-amino-5-phosphonovaleric acid to block glutamatergic transmission. CaCl_2_ in the bath was replaced with barium chloride, with the same molarity as the charge carrier, to prevent subsequent biological responses to calcium influx. **(A)** Ba^2+^ currents in a granule cell in response to the application of voltages ranging from −70 to 0 mV before (control), during (arginine vasopressin [AVP]), and after (wash) the application of AVP (0.2 nM). Subsequent application of Cd^2+^ (100 μM) and Ni^2+^ (100 μM) reduced the Ba^2+^ currents. All traces were obtained from the same cell. **(B)** Current–voltage relationships of Ba^2+^ current before and during AVP application (*n* = 11). Extracellular application of AVP significantly reduced the magnitude of Ba^2+^ currents arising in response to voltage application (*p < 0.05; **p < 0.005 vs. control, paired *t*-test. *p* = 0.7564, 0.6897, 0.2346, 0.0644, 0.0002, 0.0018, 0.0345, 0.0244 and 0.0148 at −100, −80, −60, −40, −20, 0, 20, 40 and 60 mV, respectively).

Presynaptic calcium channels represent an important target for the modulation of transmitter release in a variety of conventional nerve endings, in the central nervous system (Miller, 1998). To test the effect of AVP on presynaptic properties (i.e., granule cell activities), we examined whether AVP modulates calcium conductance in granule cells. To isolate calcium currents, voltage-clamp recordings from granule cells were made with a CsCl-based internal solution containing TEA-Cl and QX-314 (TEA^+^ internal pipette solution), where glutamatergic transmission in the bath solution was blocked by CNQX (10 μM) and AP5 (50 μM). CaCl_2_ in the bath was replaced by barium chloride with an equal molarity, used as a charge carrier to prevent subsequent biological responses to calcium influx. Figure 5A shows typical Ba^2+^ currents recorded from a granule cell evoked by voltage increasing from −70 mV (the holding membrane potential) to 0 mV. The voltage elicited an inward current that was diminished by the extracellular application of AVP. Washing out the AVP restored the evoked inward current. Subsequent application of Cd^2+^ (100 μM) and Ni^2+^ (100 μM) reduced the Ba^2+^ currents. Figure 5B shows the current–voltage (I–V) curves at the peak of the inward currents, before and during AVP application in the bath. AVP application significantly diminished the magnitude of Ba^2+^ currents in response to voltages of ≥ −40 mV. These results suggest that AVP reduces GABAergic transmission from granule to mitral cells, to some extent, through the inhibition of Ca^2+^ currents (Figure 1).

## 4 Discussion

### 4.1 Effect of AVP on dendrodendritic inhibition in the AOB

The present study demonstrated that AVP reduced the reciprocal synaptic currents (i.e., dendrodendritic inhibition) evoked in mitral cells via the activation of V1aRs. Because this reciprocal synaptic transmission encompasses glutamatergic transmission from mitral to granule cells and GABAergic transmission from granule to mitral cells, and because V1aRs are found on both mitral and granule cells in the AOB (Insel et al., 1994), both glutamatergic and GABAergic transmission should be present at the sites of action at which AVP suppresses IPSCs recorded from mitral cells. As shown in Figures 4, 5, we found that the AVP manipulation significantly suppressed voltage-activated calcium currents in granule cells, whereas extracellular application of AVP did not affect the magnitude of the response of mitral cells to GABA. This suggests that AVP reduces GABAergic transmission through the inhibition of Ca^2+^ channels on granule cells; this presynaptic mechanism is illustrated schematically in Figure 1.

Hantash et al. (2006) reported that AVP inhibited the L-type Ca^2+^ channel, which is one of the major high-voltage-activated (HVA) Ca^2+^ channels, and that its suppressive effect on that channel was prevented by a V1aR antagonist, d-(CH2)5 Tyr(Me)-AVP. The I–V relationship is shown in Figure 5, and the results suggest that AVP slightly but significantly reduced the HVA calcium currents recorded from granule cells. Thus, together with the above reports, the present results suggest that the action of AVP on dendrodendritic inhibition in the AOB can be attributed, at least partially, to the blockade of HVA calcium currents in granule cells.

As shown in Figure 5A, we tested the effect of AVP on Ca^2+^ currents of granule cells without using any antagonist or agonist for V1aRs because recording from a granule cell for more than several tens of minutes is much more difficult (than from a mitral cell, e.g., Figure 2 time plot recorded from a mitral cell). This kind of limitation for stable recordings prevented us from successively testing many conditions in a single granule cell such as control condition, application of vasopressin alone, simultaneous application of vasopressin and an antagonist for V1aR, and washing out condition. Taken the present results shown in Figures 2, 3 indicating that the vasopressin decreases dendrodendritic inhibition not via V1bR but via V1aR, suppressive effect of AVP on Ca^2+^ currents may appear through activation of V1aRs, although we were unable to directly examine the effect of the agonist/antagonist for V1aR on Ca^2+^ currents of granule cells. Further investigation using this agonist or antagonist is needed to explore the receptor subtype(s) having suppressive effect on Ca^2+^ currents of granule cells.

Concerning the effect of AVP on glutamatergic transmission from mitral to granule cells, a separate study that we conducted, to measure field excitatory postsynaptic potentials (EPSPs) evoked in granule cells and monitor the strength of mitral-to-granule glutamatergic transmission, demonstrated that AVP had no effect on basal synaptic transmission from mitral cells to granule cells (Namba et al., 2016). This result suggests that a locus for the action of AVP is GABAergic transmission from granule to mitral cells, although long-term potentiation (LTP) was induced by AVP-paired tetanic stimulation of mitral cells and blocked by the selective V1aR antagonist, Manning compound. As discussed in detail in the next section, AVP increases the excitability of mitral cells via the suppression of dendrodendritic inhibition, resulting in the induction of AVP-paired LTP at the mitral-to-granule cell synapse in the AOB. However, the tetanus-induced LTP of glutamatergic transmission in AOB slices reportedly depended on the activation of NMDA receptors (Fang et al., 2008), and AVP potentiated NMDA receptor-dependent LTP in the hippocampal CA1 region *in vivo* (Jing et al., 2009). Taken together, these observations suggest that AVP acts not only on the GABAergic transmission from granule to mitral cells as demonstrated in the present study but also on the glutamatergic transmission from mitral to granule cells through V1aRs.

### 4.2 Role of the AVP-induced reduction in dendrodendritic inhibition in pheromonal memory formation

Our separate behavioral study demonstrated that noradrenaline and the metabotropic glutamate receptor 2 (mGluR2) agonist (2S,1’R,2’R,3’R)-2-(2,3-dicarboxycyclopropyl)glycine (DCG-IV) produce selective memories in female mice of the chemosignals of male mates. These urine-based signals have the potential to prevent pregnancy if the memories are not formed (Kaba et al., 1994). Disinhibition of mitral cell activity via local infusion of an antagonist of the GABA_A_ receptor, bicuculline, creates global memories, even of the chemosignals of males other than mates. Our previous voltage-clamp studies demonstrated that activation of both the α_2_-adrenergic receptor (AR) (Huang et al., 2018) and mGluR2 (Taniguchi et al., 2013) reduced dendrodendritic inhibition of mitral cells in the mouse AOB, supporting the idea derived from the above behavioral studies that the disinhibition of mitral cells is critical for the formation of pheromonal memories (Kaba and Nakanishi, 1995). Given our finding that AVP significantly reduced dendrodendritic inhibition (Figure 2), AVP, in addition to α_2_-AR and mGluR2, may contribute to the formation of these memories.

It is conceivable that the dendrodendritic inhibition of mitral cells could act as a gateway regulating the transmission of pregnancy-blocking chemosignals. During memory formation, the association of the mate’s chemosignals with an increase in the release of noradrenaline in the AOB at the time of mating would reduce the dendrodendritic inhibition of mitral cells, resulting in increased membrane excitability of mitral cells that in turn would lead to enhanced EPSPs in granule cells (Huang et al., 2018). By suppressing GABA release to some extent during granule-to-mitral cell transmission (through partial blockade of calcium currents in granule cells), AVP and mGluR2 (Taniguchi et al., 2013) presumably provide the additional depolarization required to facilitate calcium influx through NMDA receptors, leading to NMDA receptor-dependent LTP (Fang et al., 2008) of the reciprocal synapses activated by the chemosignals of male mates. This LTP would lead to a sequential change in the morphology of the reciprocal synapses and increased release of the inhibitory neurotransmitter GABA in the AOB (Brennan et al., 1995; Matsuoka et al., 1997, 2004), resulting in the consolidation of mate recognition memories. However, the mechanism of any such process remains unknown. In fact, as mentioned in the first part of the Discussion section, not only noradrenaline (Huang et al., 2018) but also AVP (Namba et al., 2016) facilitates the induction of LTP at the glutamatergic mitral-to-granule cell synapse in the AOB, supporting this hypothesis.

After pheromonal learning, mitral cells that respond to the mate’s chemosignals would be subject to enhanced dendrodendritic inhibition due to the action of granule cells via potentiated GABAergic transmission. This enhanced inhibition of mitral cells would selectively suppress transmission of the mate’s pregnancy-blocking signals from the AOB to higher centers, such as the hypothalamus, ultimately preventing pregnancy block. Exposure to chemosignals from unfamiliar males would stimulate a different population of mitral cells, i.e., ones subject to the usual feedback inhibition; these cells would in turn send their signals to the hypothalamus, eventually provoking a neuroendocrine response leading to pregnancy loss. Disinhibition of mitral cell activity by local infusions of the GABA_A_ receptor antagonist bicuculline creates global memory in the female mice (Kaba et al., 1989). Otsuka et al. (2001) reported that disinhibition of spontaneous mitral cell activity has been observed in response to the vaginocervical stimulation. These observations support the above idea that enhancement of glutamatergic transmission from mitral to granule cells is necessary for enhanced feedback inhibition from granule to mitral cells and the formation of mate recognition memories. On the other hand, *Ex vivo* studies using targeted whole-cell recordings of identified neurons activated by the stud male in *ex vivo* brain slices from female mice demonstrated that selective reduction of mitral cell responses was due to changes in intrinsic excitability, suggesting a complementary cellular basis for encoding sensory memories in the AOB (Gao et al., 2017).

Electrophysiological studies of reciprocal synapses between mitral and granule cells in the AOB have shown that several major components influence dendrodendritic inhibition, including α_2_-ARs, NMDA receptors, mGluR1, mGluR2 (Jia et al., 1999; Taniguchi and Kaba, 2001; Castro et al., 2007; Huang et al., 2018), and V1aRs. NMDA receptors and mGluR1 enhance dendrodendritic inhibition, whereas α_2_-AR, mGluR2, and V1aRs suppress it. The complex crosstalk among these receptors regulates the excitability of mitral cells under finely tuned biological conditions, although the underlying mechanisms remain to be elucidated.

## Data Availability

The original contributions presented in the study are included in the article/supplementary material, further inquiries can be directed to the corresponding author.
